# Supplementary material for: Active twisting for adaptive droplet collection
Source: Nat Comput Sci. 2025 Apr 21;5(4):313–21. doi: 10.1038/s43588-025-00786-w (PMC12021652; doi:10.1038/s43588-025-00786-w)
Supplement: Supplementary file 1 — Details about theoretical derivations of the LCE bilayers, numerical methods and resolution techniques, discussions on mode transition and experimental methods, Supplementary Figs. 1–10 and Supplementary Tables 1 and 2. [file 43588_2025_786_MOESM1_ESM.pdf]

nature computational science

Supplementary information

https://doi.org/10.1038/s43588-025-00786-w

Active twisting for adaptive droplet collection

In the format provided by the authors and unedited

Here we provide more details about theoretical derivations of the LCE bilayers, numerical methods and resolution techniques, discussions on mode transition and experimental methods.

I. THEORIES FOR LCE BILAYERS

Many plants in nature feature ribbon-like slim leaves with a variety of morphologies, such as Persoonia helix, Pancratium maritimum, Ornithogalum concordianum, and Albuca namaquensis, as shown in Fig. 1. Essentially, the growing morphology of ribbon-like leaves is influenced by growth-induced internal stress and strain mismatches between different textural layers of tissues. Hence, we construct a LCE bilayers system to unravel the underlying mechanism and predict the growing morphogenesis.

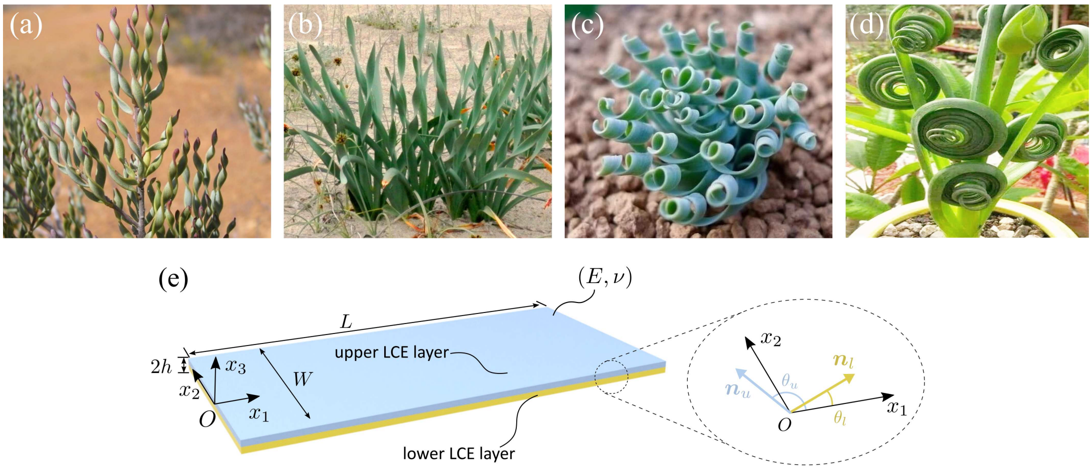

Supplementary Figure 1. Various plant morphologies. (a) The twisting shape of Persoonia helix [8]. (b) The twisting mode of Pancratium maritimum [1]. (c) The spiral shape of Ornithogalum concordianum [9]. (d) The pure bending configuration of Albuca namaquensis [10]. (e) Geometry of a LCE bilayer. The LC director $\boldsymbol{n}$ remains in x_1Ox_2 plane, with θ representing the angle between the director and x_1-axis. The subscripts l and u represent the lower and upper LCE layer, respectively.

A. A non-Euclidean shell model for monolayer system

Let us start from a shell of thickness h in the 3D Euclidean space $\mathbb{E}^3$. Any material point of the shell is described by a curvilinear coordinates as $\boldsymbol{x} = (x_1, x_2, x_3)$. The material position in the initial configuration is denoted as $\bar{\boldsymbol{r}}(\boldsymbol{x})$, while $\boldsymbol{r}(\boldsymbol{x})$ represents the current state after deformation. One can define the metric tensor $\mathrm{G}_{ij} = \boldsymbol{r}_{,i} \cdot \boldsymbol{r}_{,j}$ and $\bar{\mathrm{G}}_{ij}$ in the current and reference configuration, respectively, where the comma stands for differentiation with suffix coordinate. The Einstein's summation convention is employed throughout this paper. Without special elucidation, Latin indices $\{i, j, ...\}$ take values in $\{1, 2, 3\}$, while Greek indices $\{\alpha, \beta, ...\}$ run from $\{1, 2\}$. Using the Kirchhoff-Love assumption, the material point $\boldsymbol{r}$ can be written in terms of a normal offset from the mid-surface

$$\boldsymbol{r}(x_1, x_2, x_3) = \boldsymbol{R}(x_1, x_2) + x_3 \boldsymbol{N}(x_1, x_2), \tag{1}$$

where $\boldsymbol{R}(x_1, x_2) = \boldsymbol{r}(x_1, x_2, 0)$ and $\boldsymbol{N}$ is the unit normal of the mid-surface. The metric tensor is given by

$$\mathrm{G}_{ij} = \begin{bmatrix} \mathrm{g}_{\alpha\beta} & 0 \\ 0 & 1 \end{bmatrix}. \tag{2}$$

Substituting Eq. (1) into Eq. (2) and neglecting the higher-order terms of the thickness h, one can obtain

$$\mathrm{g}_{\alpha\beta} = \mathrm{a}_{\alpha\beta} - 2x_3 \mathrm{b}_{\alpha\beta} + \mathcal{O}(x_3^2), \tag{3}$$

in which $\mathrm{a}_{\alpha\beta}$ and $\mathrm{b}_{\alpha\beta}$ are the covariant components of the first and second fundamental forms evaluated at the mid-surface, respectively, with $\mathrm{a}_{\alpha\beta} = \boldsymbol{R}_{,\alpha} \cdot \boldsymbol{R}_{,\beta}$ and $\mathrm{b}_{\alpha\beta} = \boldsymbol{R}_{,\alpha\beta} \cdot \boldsymbol{N}$. The Green-Saint Venant strain tensor of the shell is defined as

$$\varepsilon_{\alpha\beta} = \frac{1}{2}(\mathrm{g}_{\alpha\beta} - \bar{\mathrm{g}}_{\alpha\beta}). \tag{4}$$

Combining with Eq. (3), the strain tensor (4) can be rewritten as

$$\varepsilon_{\alpha\beta} = \frac{1}{2}(\mathrm{a}_{\alpha\beta} - \bar{\mathrm{a}}_{\alpha\beta}) - x_3(\mathrm{b}_{\alpha\beta} - \bar{\mathrm{b}}_{\alpha\beta}) + \mathcal{O}(x_3^2). \tag{5}$$

Note that one can view $\Delta\mathbf{a} = (\mathbf{a} - \bar{\mathbf{a}})/2$ and $\Delta\mathbf{b} = \mathbf{b} - \bar{\mathbf{b}}$ as the membrane strain and bending strain, respectively. Considering the Saint Venant-Kirchhoff (SVK) material framework with Young's modulus E and Poisson's ratio ν, we introduce the 2D SVK material inner product as

$$\langle \mathbf{A},\ \mathbf{B} \rangle_{\mathrm{e}} := \frac{E\nu}{1-\nu^2}\mathrm{tr}(\mathbf{A})\mathrm{tr}(\mathbf{B}) + \frac{E}{1+\nu}\mathrm{tr}(\mathbf{AB}). \tag{6}$$

To simplify notations, we define the 2D SVK material norm $\|\mathbf{A}\|_{\mathrm{e}}^2 := \langle \mathbf{A},\ \mathbf{A}\rangle_{\mathrm{e}}$. The 3D elastic energy is calculated by

$$\mathcal{P} = \frac{1}{2}\int_{\bar{S}}\int_{-h/2}^{h/2} \|\bar{\mathbf{g}}^{-1}\boldsymbol{\varepsilon}\|_{\mathrm{e}}^2 \mathrm{d}x_3 \mathrm{d}\bar{S}. \tag{7}$$

We assume that only small deviations exist between the current metric $\mathbf{g}$ and the reference metric $\bar{\mathbf{g}}$, *i.e.*, $\bar{\mathbf{g}}^{-1}\mathbf{g} = \mathbf{I} + \mathcal{O}(h)$. From the definition of the 2D SVK material norm, one can obtain $\|\mathbf{A}+\mathbf{B}\|_{\mathrm{e}}^2 = \|\mathbf{A}\|_{\mathrm{e}}^2 + \|\mathbf{B}\|_{\mathrm{e}}^2 + 2\langle \mathbf{A},\ \mathbf{B}\rangle_{\mathrm{e}}$. Then, we have

$$\begin{aligned} &\bar{\mathbf{g}}^{-1}\boldsymbol{\varepsilon} = \frac{1}{2}\bar{\mathbf{a}}^{-1}\Delta\mathbf{a} - x_3\bar{\mathbf{a}}^{-1}\Delta\mathbf{b} + \mathcal{O}(x_3^2), \\ &\|\bar{\mathbf{g}}^{-1}\boldsymbol{\varepsilon}\|_{\mathrm{e}}^2 \approx \frac{1}{4}\|\bar{\mathbf{a}}^{-1}\Delta\mathbf{a}\|_{\mathrm{e}}^2 + x_3^2\|\bar{\mathbf{a}}^{-1}\Delta\mathbf{b}\|_{\mathrm{e}}^2 - x_3\left\langle \bar{\mathbf{a}}^{-1}\Delta\mathbf{a},\ \bar{\mathbf{a}}^{-1}\Delta\mathbf{b}\right\rangle_{\mathrm{e}}. \end{aligned} \tag{8}$$

Substituting Eq. (8) into Eq. (7) and integrating the energy along the thickness direction yields

$$\begin{aligned} \mathcal{P} &= \frac{1}{2}\int \left[\frac{h}{4}\|\bar{\mathbf{a}}^{-1}\Delta\mathbf{a}\|_{\mathrm{e}}^2 + \frac{h^3}{12}\|\bar{\mathbf{a}}^{-1}\Delta\mathbf{b}\|_{\mathrm{e}}^2\right]\mathrm{d}\bar{S} \\ &= \frac{1}{2}\int \left[\frac{h}{4}\|\bar{\mathbf{a}}^{-1}\Delta\mathbf{a}\|_{\mathrm{e}}^2 + \frac{h^3}{12}\|\bar{\mathbf{a}}^{-1}\Delta\mathbf{b}\|_{\mathrm{e}}^2\right]\sqrt{|\bar{\mathbf{a}}|}\mathrm{d}x_1\mathrm{d}x_2, \end{aligned} \tag{9}$$

where $|\bar{\mathbf{a}}| = \det\bar{\mathbf{a}}$. Obviously, the elastic strain energy can be decomposed into membrane and bending terms, which is analogous to the classic shell model.

B. A non-Euclidean shell model for bilayer system

We now extend the shell model to a bilayer system. We consider a shell consisting of two layers with equal thickness $H/2$. The system can be viewed as two sheets glued together at the mid-surface $x_3 = 0$. Therefore, the lower layer lies in the domain $x_3 \in [-H/2, 0]$ with the reference metric $\bar{\mathbf{g}}_l = \bar{\mathbf{a}}_l$, while the upper layer locates at $x_3 \in [0, H/2]$, denoted as $\bar{\mathbf{g}}_u = \bar{\mathbf{a}}_u$. By dividing the integral (7) into two regions, the elastic strain energy is rewritten as

$$\mathcal{P} = \frac{1}{2}\int_{\bar{S}}\int_{-H/2}^{0} \|\bar{\mathbf{g}}_l^{-1}\boldsymbol{\varepsilon}_l\|_{\mathrm{e}}^2 \mathrm{d}x_3\mathrm{d}\bar{S} + \frac{1}{2}\int_{\bar{S}}\int_{0}^{H/2} \|\bar{\mathbf{g}}_u^{-1}\boldsymbol{\varepsilon}_u\|_{\mathrm{e}}^2 \mathrm{d}x_3\mathrm{d}\bar{S}, \tag{10}$$

where $\boldsymbol{\varepsilon}_l = (\mathbf{a} - \bar{\mathbf{a}}_l)/2 - x_3\mathbf{b}$ and $\boldsymbol{\varepsilon}_u = (\mathbf{a} - \bar{\mathbf{a}}_u)/2 - x_3\mathbf{b}$ with $x_3 \in [-H/2, H/2]$. Combining Eqs. (8) and (10), we have

$$\begin{aligned} \mathcal{P} = &\frac{1}{2}\int \left[\frac{H}{8}\|\bar{\mathbf{a}}_l^{-1}\mathbf{a} - \mathbf{I}\|_{\mathrm{e}}^2 + \frac{H^3}{24}\|\bar{\mathbf{a}}_l^{-1}\mathbf{b}\|_{\mathrm{e}}^2 + \frac{H^2}{8}\left\langle \bar{\mathbf{a}}_l^{-1}\mathbf{a} - \mathbf{I},\ \bar{\mathbf{a}}_l^{-1}\mathbf{b}\right\rangle_{\mathrm{e}}\right]\sqrt{|\bar{\mathbf{a}}_l|}\mathrm{d}x_1\mathrm{d}x_2 \\ &+ \frac{1}{2}\int \left[\frac{H}{8}\|\bar{\mathbf{a}}_u^{-1}\mathbf{a} - \mathbf{I}\|_{\mathrm{e}}^2 + \frac{H^3}{24}\|\bar{\mathbf{a}}_u^{-1}\mathbf{b}\|_{\mathrm{e}}^2 - \frac{H^2}{8}\left\langle \bar{\mathbf{a}}_u^{-1}\mathbf{a} - \mathbf{I},\ \bar{\mathbf{a}}_u^{-1}\mathbf{b}\right\rangle_{\mathrm{e}}\right]\sqrt{|\bar{\mathbf{a}}_u|}\mathrm{d}x_1\mathrm{d}x_2. \end{aligned} \tag{11}$$

Note that when $\bar{\mathbf{a}}_l = \bar{\mathbf{a}}_u$, Eq. (11) reduces to (9). A question lies in how to build a connection between a bilayer and a monolayer based on the energy equivalence. Intermediate metrics [15] can be introduced,

$$\begin{aligned} \mathbf{a}_0 &= \frac{1}{2}(\bar{\mathbf{a}}_l + \bar{\mathbf{a}}_u), \\ \mathbf{b}_0 &= \frac{\zeta}{H}(\bar{\mathbf{a}}_l - \bar{\mathbf{a}}_u), \end{aligned} \tag{12}$$

where ζ is an undetermined coefficient. In prior works [2, 4], a simple choice is $\zeta = 1/2$ intuitively, which is, however, not an accurate value. To obtain a reasonable coefficient, we rewrite the above expression as

$$\begin{aligned} \bar{\mathbf{a}}_l &= \mathbf{a}_0 + \frac{H}{2\zeta}\mathbf{b}_0, \\ \bar{\mathbf{a}}_u &= \mathbf{a}_0 - \frac{H}{2\zeta}\mathbf{b}_0. \end{aligned} \tag{13}$$

Neglecting the terms of $\mathcal{O}(H)$ and higher orders, we have $\sqrt{|\mathbf{a}_0|} \approx \sqrt{|\bar{\mathbf{a}}_l|} \approx \sqrt{|\bar{\mathbf{a}}_u|}$. Besides, since the maximum principal curvature of mid-surface $\kappa_{\max}$ satisfies $\kappa_{\max} H \ll 1$ under the plane-stress approximation [7], the inverse of layer metrics can be approximated as

$$\begin{aligned} \bar{\mathbf{a}}_l^{-1} &= \left(\mathbf{a}_0 + \frac{H}{2\zeta}\mathbf{b}_0\right)^{-1} \approx \mathbf{a}_0^{-1} - \frac{H}{2\zeta}\mathbf{a}_0^{-1}\mathbf{b}_0\mathbf{a}_0^{-1}, \\ \bar{\mathbf{a}}_u^{-1} &= \left(\mathbf{a}_0 - \frac{H}{2\zeta}\mathbf{b}_0\right)^{-1} \approx \mathbf{a}_0^{-1} + \frac{H}{2\zeta}\mathbf{a}_0^{-1}\mathbf{b}_0\mathbf{a}_0^{-1}. \end{aligned} \tag{14}$$

Substituting Eq. (14) into Eq. (11), the first-order terms in Eq. (11) can be simplified as

$$\begin{aligned} \frac{H}{8}\|\bar{\mathbf{a}}_l^{-1}\mathbf{a} - \mathbf{I}\|_{\mathrm{e}}^2 &= \frac{H}{8}\|\mathbf{a}_0^{-1}\mathbf{a} - \mathbf{I}\|_{\mathrm{e}}^2 + \frac{H^3}{32\zeta^2}\|\mathbf{a}_0^{-1}\mathbf{b}_0\|_{\mathrm{e}}^2 - \frac{H^2}{8\zeta}\left\langle \mathbf{a}_0^{-1}\mathbf{a} - \mathbf{I},\ \mathbf{a}_0^{-1}\mathbf{b}_0 \right\rangle_{\mathrm{e}} + \mathcal{O}(H^4), \\ \frac{H}{8}\|\bar{\mathbf{a}}_u^{-1}\mathbf{a} - \mathbf{I}\|_{\mathrm{e}}^2 &= \frac{H}{8}\|\mathbf{a}_0^{-1}\mathbf{a} - \mathbf{I}\|_{\mathrm{e}}^2 + \frac{H^3}{32\zeta^2}\|\mathbf{a}_0^{-1}\mathbf{b}_0\|_{\mathrm{e}}^2 + \frac{H^2}{8\zeta}\left\langle \mathbf{a}_0^{-1}\mathbf{a} - \mathbf{I},\ \mathbf{a}_0^{-1}\mathbf{b}_0 \right\rangle_{\mathrm{e}} + \mathcal{O}(H^4). \end{aligned} \tag{15}$$

The second-order terms read

$$\begin{aligned} \frac{H^2}{8}\left\langle \bar{\mathbf{a}}_l^{-1}\mathbf{a} - \mathbf{I},\ \bar{\mathbf{a}}_l^{-1}\mathbf{b} \right\rangle_{\mathrm{e}} &= \frac{H^2}{8\zeta}\left\langle \mathbf{a}_0^{-1}\mathbf{a} - \mathbf{I},\ \mathbf{a}_0^{-1}\mathbf{b} \right\rangle_{\mathrm{e}} - \frac{H^3}{16\zeta}\left\langle \mathbf{a}_0^{-1}\mathbf{b}_0,\ \mathbf{a}_0^{-1}\mathbf{b} \right\rangle_{\mathrm{e}} + \mathcal{O}(H^4), \\ \frac{H^2}{8}\left\langle \bar{\mathbf{a}}_u^{-1}\mathbf{a} - \mathbf{I},\ \bar{\mathbf{a}}_l^{-1}\mathbf{b} \right\rangle_{\mathrm{e}} &= \frac{H^2}{8\zeta}\left\langle \mathbf{a}_0^{-1}\mathbf{a} - \mathbf{I},\ \mathbf{a}_0^{-1}\mathbf{b} \right\rangle_{\mathrm{e}} + \frac{H^3}{16\zeta}\left\langle \mathbf{a}_0^{-1}\mathbf{b}_0,\ \mathbf{a}_0^{-1}\mathbf{b} \right\rangle_{\mathrm{e}} + \mathcal{O}(H^4), \end{aligned} \tag{16}$$

and the third-order terms read

$$\begin{aligned} \frac{H^3}{24}\|\bar{\mathbf{a}}_l^{-1}\mathbf{b}\|_{\mathrm{e}}^2 &= \frac{H^3}{24}\|\mathbf{a}_0^{-1}\mathbf{b}\|_{\mathrm{e}}^2 + \mathcal{O}(H^4), \\ \frac{H^3}{24}\|\bar{\mathbf{a}}_u^{-1}\mathbf{b}\|_{\mathrm{e}}^2 &= \frac{H^3}{24}\|\mathbf{a}_0^{-1}\mathbf{b}\|_{\mathrm{e}}^2 + \mathcal{O}(H^4). \end{aligned} \tag{17}$$

Combining Eqs. (15)-(17) and (11), the total strain energy reads

$$\mathcal{P} = \frac{1}{2}\int \left[\frac{H}{4}\|\mathbf{a}_0^{-1}\mathbf{a} - \mathbf{I}\|_{\mathrm{e}}^2 + \frac{H^3}{16\zeta^2}\|\mathbf{a}_0^{-1}\mathbf{b}_0\|_{\mathrm{e}}^2 + \frac{H^3}{12}\|\mathbf{a}_0^{-1}\mathbf{b}\|_{\mathrm{e}}^2 - \frac{H^3}{8\zeta}\left\langle \mathbf{a}_0^{-1}\mathbf{b}_0,\ \mathbf{a}_0^{-1}\mathbf{b}\right\rangle_{\mathrm{e}}\right]\sqrt{|\mathbf{a}_0|}\mathrm{d}x_1\mathrm{d}x_2. \tag{18}$$

Obviously, when $\zeta = 3/4$, one has

$$\begin{aligned}\mathbf{a}_0 &= \frac{1}{2}(\bar{\mathbf{a}}_l + \bar{\mathbf{a}}_u),\\ \mathbf{b}_0 &= \frac{3}{4H}(\bar{\mathbf{a}}_l - \bar{\mathbf{a}}_u).\end{aligned} \tag{19}$$

The formula (18) reduces to

$$\mathcal{P} = \frac{1}{2}\int \left[\frac{H}{4}\|\mathbf{a}_0^{-1}\Delta\mathbf{a}\|_{\mathrm{e}}^2 + \frac{H^3}{12}\|\mathbf{a}_0^{-1}\Delta\mathbf{b}\|_{\mathrm{e}}^2\right]\sqrt{|\mathbf{a}_0|}\mathrm{d}x_1\mathrm{d}x_2 + \int \frac{H^3}{72}\|\mathbf{a}_0^{-1}\mathbf{b}_0\|_{\mathrm{e}}^2\sqrt{|\mathbf{a}_0|}\mathrm{d}x_1\mathrm{d}x_2, \tag{20}$$

in which $\Delta\mathbf{a} = (\mathbf{a} - \mathbf{a}_0)/2$ and $\Delta\mathbf{b} = \mathbf{b} - \mathbf{b}_0$ in the bilayer system. Note that the last term of Eq. (20) only depends on reference metrics $\bar{\mathbf{a}}_l$ and $\bar{\mathbf{a}}_u$. Therefore, Eq. (20) indicates that the bilayer structure is energetically equivalent to two monolayers with $\bar{\mathbf{a}}_l$ and $\bar{\mathbf{a}}_u$, which can be regarded as a curved monolayer with mid-surface reference fundamental forms $\mathbf{a}_0$ and $\mathbf{b}_0$.

C. LCE bilayer system

When subject to thermal load, the order degree of LC molecules decreases, leading to the spontaneous contraction along and expansion perpendicular to the LC director, which is set as uniform distribution in each LCE layer, but can be different between the two LCE layers. Here, we consider a nematic bilayer, as shown in Fig. 1(e). We establish Cartesian coordinates on the interface between two layers. Let x_1 and x_2 be mid-plane coordinates, while x_3 represents the direction perpendicular to the mean plane. We assume that the thickness, Young's modulus and Poisson's ratio of both LCE layers remain the same, denoted by $h = H/2$, E and ν, respectively. The ribbon is considered as rectangle in the x_1Ox_2 plane, with length L and width W. The two LCE layers keep constrained together at the interfaces. The general spatial orientation of LC director is denoted by a unit vector $\boldsymbol{n} = (\sin\varphi\cos\theta, \sin\varphi\sin\theta, \cos\varphi)^T$, where $\varphi_{l(u)}$ denotes the angle between director and x_3-axis, $\theta_{l(u)}$ is the angle between the projection of director on x_1Ox_2 plane and x_1-axis. The alignment of director in each layer is thus determined by two angles. The subscripts l and u represent the lower and upper LCE layer, respectively. Here, we consider the LC director

aligned in the x_1Ox_2 plane (planar alignment), *i.e.*, $\varphi_{l(u)} = 90°$, and thus the director vector is reduced to $\boldsymbol{n} = (\cos\theta, \sin\theta, 0)^T$, as shown in Fig. 1(e). We define the extension ratios in the direction parallel and perpendicular to the director are $\lambda_\parallel$ and $\lambda_\perp$, respectively, which can be expressed as

$$\begin{aligned} \lambda_\parallel &= 1 + \alpha_\parallel \Delta T, \\ \lambda_\perp &= 1 + \alpha_\perp \Delta T, \end{aligned} \tag{21}$$

where $\Delta T = T - T_0$ is the thermal change with respect to the reference temperature T_0. The spontaneous thermal expansion coefficients along the principal directions are denoted by $\alpha_\parallel$ and $\alpha_\perp$, respectively.

The reference metrics after thermal stimulation is given by

$$\bar{\mathbf{a}}_i = \mathbf{R}(\theta_i) \begin{bmatrix} \lambda_\parallel^2 & 0 \\ 0 & \lambda_\perp^2 \end{bmatrix} \mathbf{R}^T(\theta_i), \tag{22}$$

where $i = l, u$ and $\mathbf{R}(\theta_i)$ is the 2D rotation matrix. Substituting Eq. (22) into Eq. (19) yields

$$\mathbf{a}_0 = \mathbf{R}(\hat{\theta}) \begin{bmatrix} \lambda_\parallel^2 \cos^2 \frac{\Delta\theta}{2} + \lambda_\perp^2 \sin^2 \frac{\Delta\theta}{2} & 0 \\ 0 & \lambda_\perp^2 \cos^2 \frac{\Delta\theta}{2} + \lambda_\parallel^2 \sin^2 \frac{\Delta\theta}{2} \end{bmatrix} \mathbf{R}(\hat{\theta})^T, \tag{23}$$

$$\mathbf{b}_0 = \frac{3(\lambda_\parallel^2 - \lambda_\perp^2)}{4H} \mathbf{R}(\hat{\theta}) \begin{bmatrix} 0 & \sin\Delta\theta \\ \sin\Delta\theta & 0 \end{bmatrix} \mathbf{R}(\hat{\theta})^T, \tag{24}$$

where $\hat{\theta} = (\theta_u + \theta_l)/2$ is the average director angle and $\Delta\theta = \theta_u - \theta_l$ is denoted as the director angle difference. In what follows, we will analyze three distinguished deformation modes of bilayers based on the above formulations.

D. Pure bending mode

We first consider a simple case that the director of the upper layer is parallel to the x_1-axis while the lower layer is along the x_2-axis, *i.e.*, $\theta_u = 0°$ and $\theta_l = 90°$. According to Eq. (19), the equivalent fundamental forms can be expressed as

$$\begin{aligned} \mathbf{a}_0 &= \frac{1}{2}(\mathbf{a}_u + \mathbf{a}_l) = \frac{\lambda_\parallel^2 + \lambda_\perp^2}{2} \begin{bmatrix} 1 & 0 \\ 0 & 1 \end{bmatrix}, \\ \mathbf{b}_0 &= \frac{3}{4H}(\mathbf{a}_u - \mathbf{a}_l) = \frac{3\left(\lambda_\perp^2 - \lambda_\parallel^2\right)}{4H} \begin{bmatrix} 1 & 0 \\ 0 & -1 \end{bmatrix}. \end{aligned} \tag{25}$$

Since the extension ratio is close to 1, we have $\mathbf{a}_0 \sim \mathbf{I}$. The deformed configuration corresponding to Eq. (25) is a classic saddle surface. However, when $W/L \ll 1$ (as a strip), the deformation along x_2-axis can be neglected, and thus the deformed curvature satisfies

$$\frac{1}{\rho} = \frac{3\left(\lambda_\perp^2 - \lambda_\parallel^2\right)}{4H}. \tag{26}$$

Moreover, Eq. (26) can be reduced to $1/\rho = 3\left(\alpha_\perp - \alpha_\parallel\right)\Delta T/2H$ with omitting higher-order terms, which is consistent with the solution of Timoshenko's theory [14].

E. Pure twisting mode

We next consider a case that the director angles are supplementary, *i.e.*, $\theta_u + \theta_l = 180°$. The equivalent fundamental forms read

$$\begin{aligned}\mathbf{a}_0 &= \begin{bmatrix}\lambda_\perp^2 & 0\\ 0 & \lambda_\parallel^2\end{bmatrix}\cos^2\left(\frac{\Delta\theta}{2}\right) + \begin{bmatrix}\lambda_\parallel^2 & 0\\ 0 & \lambda_\perp^2\end{bmatrix}\sin^2\left(\frac{\Delta\theta}{2}\right),\\ \mathbf{b}_0 &= \frac{3\left(\lambda_\parallel^2 - \lambda_\perp^2\right)}{4H}\begin{bmatrix}0 & -\sin\Delta\theta\\ -\sin\Delta\theta & 0\end{bmatrix}.\end{aligned} \tag{27}$$

The Gaussian curvature [13] can be expressed as

$$K_G = \frac{\det\mathbf{b}_0}{\det\mathbf{a}_0} = \frac{-9\left(\lambda_\parallel^2 - \lambda_\perp^2\right)^2\sin^2\Delta\theta}{16H^2\det\mathbf{a}_0} \approx \frac{-9\left(\lambda_\perp^2 - \lambda_\parallel^2\right)^2\sin^2\Delta\theta}{16H^2}. \tag{28}$$

The parametric equation of twisting surface can be given by

$$\boldsymbol{r}(x, y, z) = \begin{cases} x = u \\ y = v\cos(ku) \\ z = v\sin(ku)\end{cases}, \tag{29}$$

where $u \in [0,\ L]$, $v \in [-W/2,\ W/2]$, and $k = 2\pi/l_w$ denotes the wave number with wavelength l_w. The first and second fundamental forms of Eq. (29) respectively read

$$\begin{aligned}\tilde{\mathbf{a}} &= \begin{bmatrix}\boldsymbol{r}_{,u}\boldsymbol{r}_{,u} & \boldsymbol{r}_{,u}\boldsymbol{r}_{,v}\\ \boldsymbol{r}_{,u}\boldsymbol{r}_{,v} & \boldsymbol{r}_{,v}\boldsymbol{r}_{,v}\end{bmatrix} = \begin{bmatrix}1 + k^2v^2 & 0\\ 0 & 1\end{bmatrix},\\ \tilde{\mathbf{b}} &= \begin{bmatrix}\boldsymbol{r}_{,uu}\boldsymbol{n} & \boldsymbol{r}_{,vu}\boldsymbol{n}\\ \boldsymbol{r}_{,uv}\boldsymbol{n} & \boldsymbol{r}_{,vv}\boldsymbol{n}\end{bmatrix} = \frac{k}{\sqrt{1 + k^2v^2}}\begin{bmatrix}0 & 1\\ 1 & 0\end{bmatrix}.\end{aligned} \tag{30}$$

Therefore, the Gaussian curvature of centerline ($v = 0$) is $\tilde{K}_G = -k^2$. Combining Eq. (28), we have

$$K_G \approx \tilde{K}_G \;\Rightarrow\; k \approx \frac{3\sin\Delta\theta\left(\alpha_\perp - \alpha_\parallel\right)\Delta T}{2H}. \tag{31}$$

Obviously, when $\Delta\theta = 90^\circ$, the wave number k reaches its maximum value. Note that our theory covers the prior prediction in [4], where the twisting deformation occurs only at $\theta_u = 45^\circ$ and $\theta_l = 135^\circ$, while our results suggest a much wider range of solution group satisfying $\theta_u + \theta_l = 180^\circ$.

F. Bending-twisting coupling spiral shape

We now discuss a more general case of bending-twisting coupling spiral mode. Let κ_1 and κ_2 be the principal curvatures of the mid-surface, the Gaussian curvature can be expressed as $K_G = \kappa_1\kappa_2$ and mean curvature reads $H_{avg} = (\kappa_1 + \kappa_2)/2$. With the assumption $\mathbf{a}_0 \sim \mathbf{I}$, we have $K_g \approx \det \mathbf{b}_0$ and $H_{avg} \approx \mathrm{tr}(\mathbf{b}_0)/2$. Therefore, the principal curvature κ_1 and κ_2 can be seen as the eigenvalues of $\mathbf{b}_0$. To obtain κ_1 and κ_2, we first choose a matrix

$$\mathbf{A_0} = \begin{bmatrix} 0 & 1 \\ 1 & 0 \end{bmatrix}, \tag{32}$$

where the eigenvalues λ_1 and λ_2, and eigenvectors $\boldsymbol{t}_1$ and $\boldsymbol{t}_2$ read

$$\begin{bmatrix} \lambda_1 \\ \lambda_2 \end{bmatrix} = \begin{bmatrix} 1 \\ -1 \end{bmatrix}, \quad \begin{bmatrix} \boldsymbol{t}_1 & \boldsymbol{t}_2 \end{bmatrix} = \frac{1}{2}\begin{bmatrix} \sqrt{2} & -\sqrt{2} \\ \sqrt{2} & \sqrt{2} \end{bmatrix} = \mathbf{R}\left(\frac{\pi}{4}\right). \tag{33}$$

After any in-plane rotation of the coordinate system, the matrix is transformed into $\mathbf{A}_R = \mathbf{R}\left(\hat{\theta}\right)\mathbf{A}_0\mathbf{R}\left(\hat{\theta}\right)^T$, we have

$$\begin{aligned} \mathbf{A}_R \cdot \left(\mathbf{R}\left(\hat{\theta}\right)\boldsymbol{t}_1\right) &= \mathbf{R}\left(\hat{\theta}\right)\mathbf{A}_0\mathbf{R}\left(\hat{\theta}\right)^T\mathbf{R}\left(\hat{\theta}\right)\boldsymbol{t}_1 = \mathbf{R}\left(\hat{\theta}\right)\mathbf{A}_0\boldsymbol{t}_1 = \lambda_1\mathbf{R}\left(\hat{\theta}\right)\boldsymbol{t}_1, \\ \mathbf{A}_R \cdot \left(\mathbf{R}\left(\hat{\theta}\right)\boldsymbol{t}_2\right) &= \mathbf{R}\left(\hat{\theta}\right)\mathbf{A}_0\mathbf{R}\left(\hat{\theta}\right)^T\mathbf{R}\left(\hat{\theta}\right)\boldsymbol{t}_2 = \mathbf{R}\left(\hat{\theta}\right)\mathbf{A}_0\boldsymbol{t}_2 = \lambda_2\mathbf{R}\left(\hat{\theta}\right)\boldsymbol{t}_2. \end{aligned} \tag{34}$$

Obviously, the eigenvectors and eigenvalues of $\mathbf{A}_R$ respectively read

$$\begin{bmatrix} \boldsymbol{q}_1 & \boldsymbol{q}_2 \end{bmatrix} = \mathbf{R}\left(\hat{\theta}\right)\mathbf{R}\left(\frac{\pi}{4}\right) = \mathbf{R}\left(\hat{\theta} + \frac{\pi}{4}\right), \quad \begin{bmatrix} \lambda_1 \\ \lambda_2 \end{bmatrix} = \begin{bmatrix} 1 \\ -1 \end{bmatrix}. \tag{35}$$

We rewrite Eq. (24) as $\mathbf{b} = b_0 \mathbf{A}_R$ and thus the corresponding eigenvalues and unit eigenvectors are

$$\begin{bmatrix} \kappa_1 \\ \kappa_2 \end{bmatrix} = b_0 \begin{bmatrix} 1 \\ -1 \end{bmatrix}, \quad \begin{bmatrix} \boldsymbol{q}_1 & \boldsymbol{q}_2 \end{bmatrix} = \mathbf{R}\left(\hat{\theta} + \frac{\pi}{4}\right), \tag{36}$$

where $\boldsymbol{q}_1$ and $\boldsymbol{q}_2$ are the unit eigenvectors corresponding to κ_1 and κ_2, and $b_0 = 3(\lambda_\parallel^2 - \lambda_\perp^2)\sin\Delta\theta/4H$. Note that $\boldsymbol{q}_1$ and $\boldsymbol{q}_2$ indicate the directions of principal curvature as well.

The angle between the principal direction and the coordinate system is denoted as $\phi = \hat{\theta} + \pi/4$. Then, the helix angle $\varPhi$ and radius R can be determined by [6]

$$\begin{aligned} \varPhi &= \arctan \frac{(\kappa_1 - \kappa_2)\sin\phi\cos\phi}{\kappa_1\cos^2\phi + \kappa_2\sin^2\phi}, \\ R &= \frac{\kappa_1\cos^2\phi + \kappa_2\sin^2\phi}{\kappa_1^2\cos^2\phi + \kappa_2^2\sin^2\phi}. \end{aligned} \tag{37}$$

Combining Eq. (36), Eq. (37) is reduced to

$$\begin{aligned} \varPhi &= \arctan \frac{2\sin\phi\cos\phi}{\cos^2\phi - \sin^2\phi} = 2\phi, \\ R &= \frac{b_0\cos^2\phi - b_0\sin^2\phi}{b_0^2\cos^2\phi + b_0^2\sin^2\phi} = \frac{\cos 2\phi}{b_0}. \end{aligned} \tag{38}$$

Note that the helix angle only depends on the director orientation and is irrelevant to thermal change.

G. Bending stiffness of a twisted ribbon

Experiments suggest that twisting configuration exhibits superior wind resistance because of high bending stiffness. We now analytically derive the bending stiffness of a twisted ribbon as shown in Fig. I G, where the Cartesian coordinate system is employed. The origin O is located at the centroid of the cross section of the ribbon, with the x_1, x_2, and x_3 axes along with the directions of length L, width W, and thickness H, respectively. A twisted coordinate system $(\hat{x}_1, \hat{x}_2, \hat{x}_3)$ is introduced, which is fixed to the cross section of the ribbon and rotates with the twisting angle. Note that the axes $\hat{x}_2$ and $\hat{x}_3$ are in the principal directions of the cross section.

Denoting the total twisting angle of the beam as β and introducing $\bar{\beta} = \beta/L$ to represent the twisting angle per unit length, the area moments of inertia I_{22} and I_{33}, and the polar

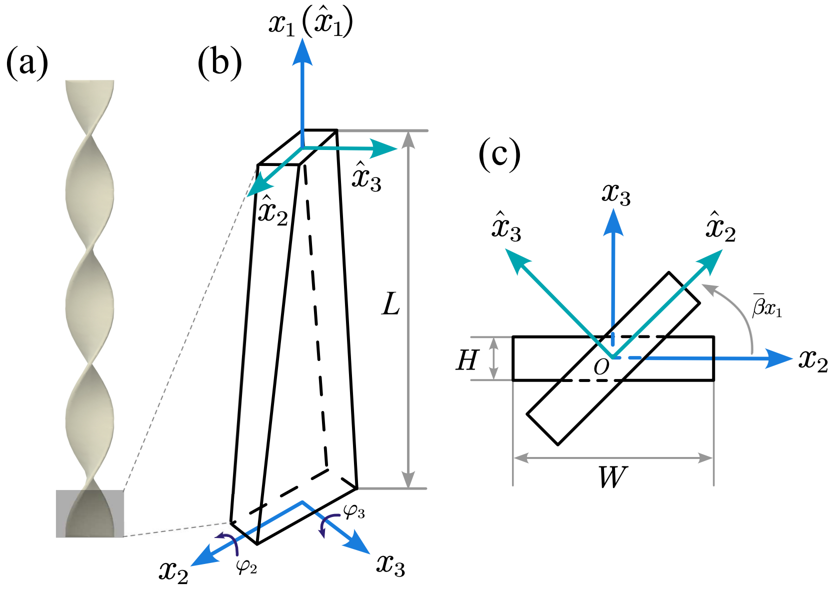

Supplementary Figure 2. Geometry of a twisted ribbon. (a) Schematic diagram of the twisted mode. (b) Global coordinate system (x_1, x_2, x_3) and local coordinate system $(\hat{x}_1, \hat{x}_2, \hat{x}_3)$. (c) Global and local coordinate systems in the cross-sectional view.

moment of area I_{23} are expressed as

$$\begin{aligned} I_{22} &= I_2 \cos^2\left(\bar{\beta}x_1\right) + I_3 \sin^2(\bar{\beta}x_1), \\ I_{33} &= I_2 \sin^2(\bar{\beta}x_1) + I_3 \cos^2(\bar{\beta}x_1), \\ I_{23} &= (I_3 - I_2)\sin(\bar{\beta}x_1)\cos(\bar{\beta}x_1), \end{aligned} \tag{39}$$

where $I_2 = WH^3/12$ and $I_3 = W^3H/12$ denote the principal area moments of inertia of the cross section.

According to Timoshenko beam theory, u_2 and u_3 represent the deflections of the centroid of cross section, while φ_2 and φ_3 represent the rotations of the cross section. The normal strain is denoted as

$$\varepsilon_{11} = x_2 \frac{\mathrm{d}\varphi_3}{\mathrm{d}x_1} + x_3 \frac{\mathrm{d}\varphi_2}{\mathrm{d}x_1}. \tag{40}$$

Assuming the material is linearly elastic and isotropic, the bending moments M_2 and M_3 along the x_2 and x_3 directions on the cross section can be expressed as

$$\begin{aligned} M_2 &= \iint_A E\varepsilon_{11} x_3 \mathrm{d}A = EI_{22}\frac{\mathrm{d}\varphi_2}{\mathrm{d}x_1} + EI_{23}\frac{\mathrm{d}\varphi_3}{\mathrm{d}x_1}, \\ M_3 &= \iint_A E\varepsilon_{11} x_2 \mathrm{d}A = EI_{23}\frac{\mathrm{d}\varphi_2}{\mathrm{d}x_1} + EI_{33}\frac{\mathrm{d}\varphi_3}{\mathrm{d}x_1}, \end{aligned} \tag{41}$$

where E is the Young's modulus and A denotes the cross-sectional area of the ribbon. The equilibrium equations are derived by the minimization of the total potential energy Π_t,

$$\delta\Pi_t = \delta\left(U + V_f\right) = 0, \tag{42}$$

in which $U = U_b + U_s$ is the internal strain energy of the ribbon due to bending and shearing, and U_b and U_s can be respectively calculated by

$$\begin{aligned} U_\mathrm{b} &= \frac{1}{2}\int_0^L \left[EI_{22}\left(\frac{\mathrm{d}\varphi_2}{\mathrm{d}x_1}\right)^2 + 2EI_{23}\frac{\mathrm{d}\varphi_2}{\mathrm{d}x_1}\frac{\mathrm{d}\varphi_3}{\mathrm{d}x_1} + EI_{33}\left(\frac{\mathrm{d}\varphi_3}{\mathrm{d}x_1}\right)^2\right]\mathrm{d}x_1, \\ U_\mathrm{s} &= \frac{1}{2}\int_0^L \omega GA\left[\left(\frac{\mathrm{d}u_2}{\mathrm{d}x_1} - \varphi_3\right)^2 + \left(\frac{\mathrm{d}u_3}{\mathrm{d}x_1} - \varphi_2\right)^2\right]\mathrm{d}x_1, \end{aligned} \tag{43}$$

where $G = E/[2(1+\nu)]$ is the shear modulus and $\omega = 0.83$ is a shearing correction factor for rectangular ribbons [17].

The external work V_f of the uniformly distributed transverse force f from the wind in the x_3 direction is written as

$$V_f = -\int_0^L f u_3 \mathrm{d}x_1. \tag{44}$$

Substituting Eqs. (43) and (44) into (42) leads to a set of static equilibrium equations,

$$\begin{aligned} &\frac{\mathrm{d}^2 u_2}{\mathrm{d}x_1^2} = \frac{\mathrm{d}\varphi_3}{\mathrm{d}x_1}, \\ &\frac{\mathrm{d}^2 u_3}{\mathrm{d}x_1^2} = -\frac{f}{\omega GA} + \frac{\mathrm{d}\varphi_2}{\mathrm{d}x_1}, \\ &\frac{\mathrm{d}}{\mathrm{d}x_1}\left(I_{22}\frac{\mathrm{d}\varphi_2}{\mathrm{d}x_1} + I_{23}\frac{\mathrm{d}\varphi_3}{\mathrm{d}x_1}\right) = \frac{\omega GA}{E}\left(\varphi_2 - \frac{\mathrm{d}u_3}{\mathrm{d}x_1}\right), \\ &\frac{\mathrm{d}}{\mathrm{d}x_1}\left(I_{23}\frac{\mathrm{d}\varphi_2}{\mathrm{d}x_1} + I_{33}\frac{\mathrm{d}\varphi_3}{\mathrm{d}x_1}\right) = \frac{\omega GA}{E}\left(\varphi_3 - \frac{\mathrm{d}u_2}{\mathrm{d}x_1}\right), \end{aligned} \tag{45}$$

with boundary conditions,

$$u_2|_{x_1=0} = u_3|_{x_1=0} = \varphi_2|_{x_1=0} = \varphi_3|_{x_1=0} = 0, \tag{46}$$

$$\begin{aligned} &\omega GA\left(\frac{\mathrm{d}u_2}{\mathrm{d}x_1} - \varphi_3\right)\bigg|_{x_1=L} = \omega GA\left(\frac{\mathrm{d}u_3}{\mathrm{d}x_1} - \varphi_2\right)\bigg|_{x_1=L} = 0, \\ &\left(EI_{22}\frac{\mathrm{d}\varphi_2}{\mathrm{d}x_1} + EI_{23}\frac{\mathrm{d}\varphi_3}{\mathrm{d}x_1}\right)\bigg|_{x_1=L} = \left(EI_{33}\frac{\mathrm{d}\varphi_3}{\mathrm{d}x_1} + EI_{23}\frac{\mathrm{d}\varphi_2}{\mathrm{d}x_1}\right)\bigg|_{x_1=L} = 0. \end{aligned} \tag{47}$$

Considering the boundary conditions (46) and (47), the static equilibrium equations in (45)

transform into

$$\begin{aligned}
&\frac{\mathrm{d}^2 u_2}{\mathrm{d}x_1^2} = \frac{(I_2 - I_3)\sin(2\bar{\beta}x_1)}{4I_2I_3E} f(L-x_1)^2,\\
&u_2|_{x_1=0} = 0, \quad \left.\frac{\mathrm{d}u_2}{\mathrm{d}x_1}\right|_{x_1=0} = 0,\\
&\frac{\mathrm{d}^2 u_3}{\mathrm{d}x_1^2} = -\frac{f}{\omega GA} + \frac{I_2\sin^2(\bar{\beta}x_1) + I_3\cos^2(\bar{\beta}x_1)}{2I_2I_3E} f(L-x_1)^2,\\
&u_3|_{x_1=0} = 0, \quad \left.\frac{\mathrm{d}u_3}{\mathrm{d}x_1}\right|_{x_1=0} = \frac{fL}{\omega GA}.
\end{aligned} \tag{48}$$

Through these two differential equations and four boundary conditions, deflections in the x_2 and x_3 directions can be derived as

$$u_2 = \frac{fC_2(I_2 - I_3)}{16\bar{\beta}^4 EI_2I_3}, \quad u_3 = \frac{fC_3}{96\bar{\beta}^4 EI_3}. \tag{49}$$

The expressions for C_2 and C_3 are given by [17]

$$\begin{aligned}
C_2 = &\left[\frac{3}{2} - (\beta-\gamma)^2\right]\sin 2\gamma + 2(\beta-\gamma)\cos 2\gamma\\
&- 2\beta + \left(2\beta^2 - 1\right)\gamma,\\
C_3 = &48\eta\beta^2\gamma(2\beta-\gamma) + 2\gamma^2\left(1+\mu^2\right)\left(6\beta^2 - 4\beta\gamma + \gamma^2\right)\\
&+ 3\left(1-\mu^2\right)\left\{\left[2(\beta-\gamma)^2 - 3\right]\cos 2\gamma\right.\\
&\left.+4(\beta-\gamma)\sin 2\gamma + 3 - 2\beta^2 - 4\beta\gamma\right\},
\end{aligned} \tag{50}$$

where

$$\mu = \frac{W}{H}, \quad \gamma = \bar{\beta}x_1, \quad \eta = \frac{EI_3}{\omega GAL^2}. \tag{51}$$

Let $k_t = f/u$ be the bending stiffness, where $u = \sqrt{{u_2}^2 + {u_3}^2}$ denotes the total deflection. Using EI_3/L^4 for nondimensionalization of k_t leads to

$$\bar{k} = \frac{96\beta^4 I_2}{\sqrt{36{C_2}^2\left(I_2 - I_3\right)^2 + {C_3}^2{I_2}^2}}. \tag{52}$$

As shown in Fig. 4(j) in the main text, with the increase of twisting angle, the bending stiffness dramatically increases, which suggests that the twisting configuration provides a significant advantage in wind resistance.

II. A COMPUTATIONAL MODEL OF LCE BILAYERS

We next develop a computational model of LCE bilayers to predict their morphology evolutions and transitions. Similar to the classical kinematics of solid-shell geometry [16],

deformation of a LCE strip can be described by material points at the top and bottom surfaces of the shell. Therefore, the position vector of a material point in the initial (reference) configuration $\mathfrak{B}_0$ reads

$$\bar{\boldsymbol{r}}(\xi^1,\xi^2,\xi^3) = \frac{1}{2}(1+\xi^3)\bar{\boldsymbol{r}}_{\mathrm{T}}(\xi^1,\xi^2) + \frac{1}{2}(1-\xi^3)\bar{\boldsymbol{r}}_{\mathrm{B}}(\xi^1,\xi^2), \tag{53}$$

where (ξ^1,ξ^2,ξ^3) are natural coordinates ranging from $[-1,1]$, and subscripts T and B denote the top and bottom surfaces, respectively. Similarly, the vector $\boldsymbol{r}$ in the deformed (current) configuration $\mathfrak{B}_t$ is given by

$$\boldsymbol{r}(\xi^1,\xi^2,\xi^3) = \frac{1}{2}(1+\xi^3)\boldsymbol{r}_{\mathrm{T}}(\xi^1,\xi^2) + \frac{1}{2}(1-\xi^3)\boldsymbol{r}_{\mathrm{B}}(\xi^1,\xi^2). \tag{54}$$

Therefore, the displacement $\boldsymbol{u}$ can be expressed as

$$\boldsymbol{u}(\xi^1,\xi^2,\xi^3) = \boldsymbol{r}(\xi^1,\xi^2,\xi^3) - \bar{\boldsymbol{r}}(\xi^1,\xi^2,\xi^3). \tag{55}$$

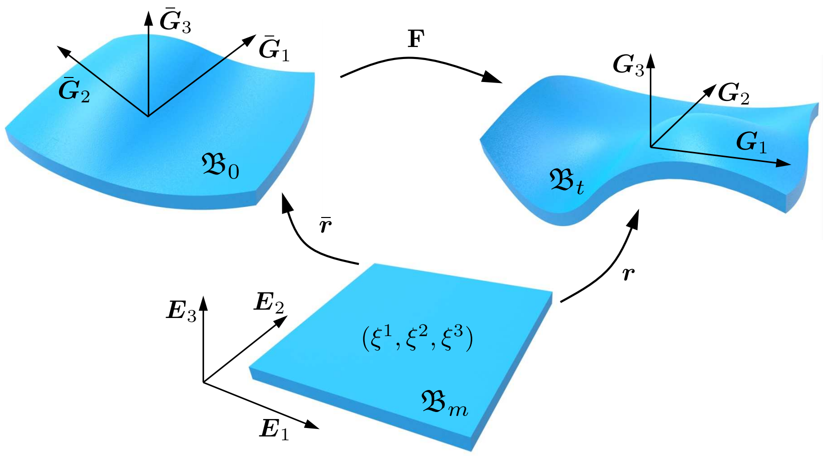

Supplementary Figure 3. The three configurations of a shell, distinguished as the reference configuration $\mathfrak{B}_0$, current configuration $\mathfrak{B}_t$ and parametric configuration $\mathfrak{B}_m$.

The deformation gradient reads

$$\mathbf{F} = \frac{\partial \boldsymbol{r}}{\partial \bar{\boldsymbol{r}}} = \boldsymbol{G}_i \otimes \bar{\boldsymbol{G}}^i, \tag{56}$$

where convected basis vectors in the reference configuration read $\bar{\boldsymbol{G}}_i := \partial\bar{\boldsymbol{r}}/\partial\xi^i,\ i=1,2,3$, and in the current configuration read $\boldsymbol{G}_i := \partial\boldsymbol{r}/\partial\xi^i,\ i=1,2,3$.

The Green-Lagrange strain tensor reads

$$\mathbf{E} = \frac{1}{2}(\mathbf{F}^T\mathbf{F} - \mathbf{I}) = \frac{1}{2}(\mathrm{G}_{ij} - \bar{\mathrm{G}}_{ij})\bar{\boldsymbol{G}}^i \otimes \bar{\boldsymbol{G}}^j = \mathrm{E}_{ij}\bar{\boldsymbol{G}}^i \otimes \bar{\boldsymbol{G}}^j. \tag{57}$$

Combining with Eq. (55), the components of metric tensor G_{ij} can be written as

$$\mathrm{G}_{ij} = \boldsymbol{G}_i \cdot \boldsymbol{G}_j = (\bar{\boldsymbol{G}}_i + \frac{\partial \boldsymbol{u}}{\partial \xi^i}) \cdot (\bar{\boldsymbol{G}}_j + \frac{\partial \boldsymbol{u}}{\partial \xi^j}) = \bar{\mathrm{G}}_{ij} + \bar{\boldsymbol{G}}_i \cdot \frac{\partial \boldsymbol{u}}{\partial \xi^j} + \frac{\partial \boldsymbol{u}}{\partial \xi^i} \cdot \bar{\boldsymbol{G}}_j + \frac{\partial \boldsymbol{u}}{\partial \xi^i} \cdot \frac{\partial \boldsymbol{u}}{\partial \xi^j}. \quad (58)$$

Substituting Eq. (58) into Eq. (57) yields the Green-Lagrange strain tensor

$$\mathrm{E}_{ij} = \frac{1}{2}(\bar{\boldsymbol{G}}_i \cdot \frac{\partial \boldsymbol{u}}{\partial \xi^j} + \frac{\partial \boldsymbol{u}}{\partial \xi^i} \cdot \bar{\boldsymbol{G}}_j + \frac{\partial \boldsymbol{u}}{\partial \xi^i} \cdot \frac{\partial \boldsymbol{u}}{\partial \xi^j}). \quad (59)$$

Note that in Eq. (59), the Kirchhoff hypothesis is removed and the higher-order term of ξ^3 is reserved. With the spontaneous strains of LCE in the direction parallel and perpendicular to the director $\mathrm{E}^s_{\parallel} = \lambda_{\parallel} - 1$ and $\mathrm{E}^s_{\perp} = \lambda_{\perp} - 1$, the spontaneous strain tensor can be expressed as

$$\mathbf{E}^s = \mathrm{E}^s_{\perp}\mathbf{I} + \left(\mathrm{E}^s_{\parallel} - \mathrm{E}^s_{\perp}\right) \boldsymbol{n} \otimes \boldsymbol{n}, \quad (60)$$

where $\mathbf{I}$ represents the identity tensor, and unit vector $\boldsymbol{n}$ denotes the orientation of LC director. Substituting $\boldsymbol{n}$ into Eq. (60) yields the matrix form of spontaneous strain tensor,

$$[\mathrm{E}^s] = \begin{bmatrix} \mathrm{E}^s_{\perp} + \left(\mathrm{E}^s_{\parallel} - \mathrm{E}^s_{\perp}\right)\sin^2\varphi\cos^2\theta & \left(\mathrm{E}^s_{\parallel} - \mathrm{E}^s_{\perp}\right)\sin^2\varphi\sin 2\theta & \left(\mathrm{E}^s_{\parallel} - \mathrm{E}^s_{\perp}\right)\sin 2\varphi\cos\theta \\ & \mathrm{E}^s_{\perp} + \left(\mathrm{E}^s_{\parallel} - \mathrm{E}^s_{\perp}\right)\sin^2\varphi\sin^2\theta & \left(\mathrm{E}^s_{\parallel} - \mathrm{E}^s_{\perp}\right)\sin 2\varphi\sin\theta \\ \text{sym.} & & \mathrm{E}^s_{\parallel}\cos^2\varphi + \mathrm{E}^s_{\perp}\sin^2\varphi \end{bmatrix}, \quad (61)$$

in which the engineering shear strains $\gamma^s_{ij} = 2\mathrm{E}^s_{ij}$ $(i \neq j)$ are used. For the upper or lower LCE layer, the components of spontaneous strains can be calculated when the subscripts of the angles in Eq. (61) are taken as u or l. When the LC director is aligned in the x_1Ox_2 plane (planar alignment), one can derive the components of spontaneous strain as

$$[\mathrm{E}^s] = \begin{bmatrix} \mathrm{E}^s_{\perp}\sin^2\theta + \mathrm{E}^s_{\parallel}\cos^2\theta & \left(\mathrm{E}^s_{\parallel} - \mathrm{E}^s_{\perp}\right)\sin 2\theta & 0 \\ & \mathrm{E}^s_{\perp}\cos^2\theta + \mathrm{E}^s_{\parallel}\sin^2\theta & 0 \\ \text{sym.} & & \mathrm{E}^s_{\perp} \end{bmatrix}. \quad (62)$$

The elastic strain can be expressed as $\mathbf{E}^e = \mathbf{E} - \mathbf{E}^s$, and the second Piola-Kirchhoff stress tensor $\mathbf{S}$ can be given by

$$\mathbf{S} = \frac{\partial W_e}{\partial \mathbf{E}^e}, \quad (63)$$

where W_e is the stored strain energy. Note that $\mathbf{S}$ is conjugated with the Green-Lagrange strain $\mathbf{E}$. Using the basis $\bar{\boldsymbol{G}}_i \otimes \bar{\boldsymbol{G}}_j$, $\mathbf{S}$ can be written as

$$\mathbf{S} = \mathrm{S}^{ij}\bar{\boldsymbol{G}}_i \otimes \bar{\boldsymbol{G}}_j, \quad (64)$$

where S^{ij} are its contravariant components. Since the elastic strain remains small in the LCE strip, we consider the Saint-Venant Kirchhoff (SVK) constitutive law, and thus the second Piola-Kirchhoff stress tensor $\mathbf{S}$ reads

$$\mathbf{S} = \lambda \mathrm{tr}(\mathbf{E}^e)\bar{\boldsymbol{G}}_i \otimes \bar{\boldsymbol{G}}_j + 2\mu\mathbf{E}^e, \tag{65}$$

where $\lambda = E\nu/[(1+\nu)(1-2\nu)]$ and $\mu = E/[2(1+\nu)]$ denote Lamé's material constants.

Based on the above formulations, one can predict the morphology evolution of LCE bilayers. However, for large deformations of thin-walled structures, numerical locking problems may occur in computation, leading to inaccurate predictions. In what follows, we implement a simple low-order solid-shell element formulation to remedy this issue.

A. EAS and ANS formulation

We provide a brief description of enhanced assumed strain (EAS) method [11] to relieve the Poisson-thickness locking and volumetric locking problems. Compared to the enhancement of the deformation gradient $\mathbf{F}$, we choose an alternative way by enhancing the Green-Lagrange strain $\mathbf{E}$, which leads to the same result yet more simple and effective [16]. The enhanced elastic strain $\mathbf{E}^e$ and its variation $\delta\mathbf{E}^e$ can be defined as

$$\mathbf{E}^e = \mathbf{E} - \mathbf{E}^s + \tilde{\mathbf{E}}, \tag{66}$$

$$\delta\mathbf{E}^e = \delta\mathbf{E} + \delta\tilde{\mathbf{E}}, \tag{67}$$

where $\tilde{\mathbf{E}}$ and $\delta\tilde{\mathbf{E}}$ represent the enhancing strain and its variation. The modified three-field Fraeijs de Veubeke-Hu-Washizu (FHW) variational principle is given by [12]

$$\begin{aligned}\Pi(\boldsymbol{u}, \tilde{\mathbf{E}}, \mathbf{S}) = &\int_{\mathfrak{B}_0} W_e(\mathbf{E}(\boldsymbol{u}) - \mathbf{E}^s + \tilde{\mathbf{E}})\mathrm{d}V - \int_{\mathfrak{B}_0} \mathbf{S} : \tilde{\mathbf{E}}\mathrm{d}V \\ &- \int_{\mathfrak{B}_0} \boldsymbol{u}\cdot\boldsymbol{b}^*\mathrm{d}V - \int_{\partial\mathfrak{B}_0} \boldsymbol{u}\cdot\boldsymbol{t}^*\mathrm{d}S,\end{aligned} \tag{68}$$

in which the displacement $\boldsymbol{u}$, the enhanced assumed strain tensor $\tilde{\mathbf{E}}$ and the second Piola−Kirchhoff stress tensor $\mathbf{S}$ are all independent variables. The known body force is denoted by $\boldsymbol{b}^*$, while $\boldsymbol{t}^*$ is the prescribed traction on the boundary $\partial\mathfrak{B}_0$. All variables here are expressed in the reference configuration $\mathfrak{B}_0$.

The main purpose of the EAS lies in designing the stress field $\mathbf{S}$ and enhancing strain $\tilde{\mathbf{E}}$ that satisfy the following orthogonality condition,

$$\int_{\mathfrak{B}_0} \mathbf{S} : \tilde{\mathbf{E}} \mathrm{d}V = 0. \tag{69}$$

Considering Eqs. (68) and (69), the corresponding variational form can be written as

$$\begin{aligned} &\int_{\mathfrak{B}_0} \delta\mathbf{E} : \mathbf{S} \mathrm{d}V = \int_{\mathfrak{B}_0} \delta\boldsymbol{u} \cdot \boldsymbol{b}^* \mathrm{d}V + \int_{\partial\mathfrak{B}_0} \delta\boldsymbol{u} \cdot \boldsymbol{t}^* \mathrm{d}S, \\ &\int_{\mathfrak{B}_0} \delta\tilde{\mathbf{E}} : \mathbf{S} \mathrm{d}V = 0. \end{aligned} \tag{70}$$

B. Spatial discretization

In the finite element simulation, the reference configuration $\mathfrak{B}_0$ is discretized into n non-overlapping elements $\mathfrak{B}_0^{(e)}$. The tangent matrix in the Newton-Raphson solution procedure is constructed by taking the directional derivative of the weak form (70). Here, we use the eight-node brick element for discretization. The solid-shell element only contains displacement degrees of freedom (DOFs), and does not need a sophisticated finite-rotation update and transition elements to link solid-shell elements to conventional solid elements. The displacement field and its increment with the shape function matrix $\mathbb{N}$ can be interpolated as

$$\boldsymbol{u} = \mathbb{N}\boldsymbol{d}^{(e)}, \quad \Delta\boldsymbol{u} = \mathbb{N}\Delta\boldsymbol{d}^{(e)}, \tag{71}$$

where $\boldsymbol{d}^{(e)}$ and $\Delta\boldsymbol{d}^{(e)}$ are the vectors of nodal displacement and its increment, respectively. Using the Voigt notation, the vectorized Green-Lagrange strain becomes

$$\{\mathbf{E}\} = \mathbf{B}\boldsymbol{d}^{(e)}, \quad \{\mathbf{E}\} = [\mathrm{E}_{11}\ \mathrm{E}_{22}\ \mathrm{E}_{33}\ 2\mathrm{E}_{12}\ 2\mathrm{E}_{23}\ 2\mathrm{E}_{13}]^T, \tag{72}$$

in which $\mathbf{B}$ is the strain-displacement matrix.

We employ the 7-parameter enhanced strain $\tilde{\mathbf{E}}$, in which three parameters on the transverse normal strain are used, to remedy the Poisson-thickness locking phenomenon. Besides, in hyperelastic or elastoplastic analyses, volumetric locking is often a critical problem. Thus, additional four EAS parameters for the in-plane normal strains are also introduced to alleviate the volumetric locking and to enhance the membrane bending behavior. We compute the enhanced strain $\tilde{\mathbf{E}}$ and its variant by

$$\{\tilde{\mathrm{E}}_{ij}\} = \mathbf{M}(\boldsymbol{\xi})\boldsymbol{\alpha}^{(e)}, \quad \{\delta\tilde{\mathrm{E}}_{ij}\} = \mathbf{M}(\boldsymbol{\xi})\delta\boldsymbol{\alpha}^{(e)}, \tag{73}$$

where $\mathbf{M}$ is the trial function matrix of the enhancing strain, $\boldsymbol{\alpha}$ denotes the vector of EAS parameters. The enhanced Green−Lagrange strain tensor can be interpolated all over the element domain using the vectors $\boldsymbol{d}$ and the enhancing strain parameter,

$$\mathbf{E}^e = \mathbf{E} + \tilde{\mathbf{E}} - \mathbf{E}^s = \begin{bmatrix} \mathbf{B} & \mathbf{M} \end{bmatrix} \begin{bmatrix} \boldsymbol{d} \\ \boldsymbol{\alpha} \end{bmatrix} - \mathbf{E}^s, \tag{74}$$

in which $\mathbf{M}$ needs to be transformed into the physical Cartesian coordinate system

$$\mathbf{M} = \frac{J_0}{J}\mathbf{T}_0\tilde{\mathbf{M}}, \tag{75}$$

where $\tilde{\mathbf{M}}$ is the trial function of the enhancing strain in physical Cartesian coordinate system, J_0 and J are the determinants of the Jacobian matrices at the element center and at any arbitrary point in the element domain, respectively. The transformation matrix $\mathbf{T}_0$ reads [16]

$$\mathbf{T}_0 = \begin{bmatrix} (a_1^1)^2 & (a_1^2)^2 & (a_1^3)^2 & a_1^1 a_1^2 & a_1^2 a_1^3 & a_1^3 a_1^1 \\ (a_2^1)^2 & (a_2^2)^2 & (a_2^3)^2 & a_2^1 a_2^2 & a_2^2 a_2^3 & a_2^3 a_2^1 \\ (a_3^1)^2 & (a_3^2)^2 & (a_3^3)^2 & a_3^1 a_3^2 & a_3^2 a_3^3 & a_3^3 a_3^1 \\ 2a_1^1 a_2^1 & 2a_1^2 a_2^2 & 2a_1^3 a_2^3 & a_1^1 a_2^2 + a_2^1 a_1^2 & a_1^2 a_2^3 + a_2^2 a_1^3 & a_1^3 a_2^1 + a_2^3 a_1^1 \\ 2a_2^1 a_3^1 & 2a_2^2 a_3^2 & 2a_2^3 a_3^3 & a_2^1 a_3^2 + a_3^1 a_2^2 & a_2^2 a_3^3 + a_3^2 a_2^3 & a_2^3 a_3^1 + a_3^3 a_2^1 \\ 2a_3^1 a_1^1 & 2a_3^2 a_1^2 & 2a_3^3 a_1^3 & a_3^1 a_1^2 + a_1^1 a_3^2 & a_3^2 a_1^3 + a_1^2 a_3^3 & a_3^3 a_1^1 + a_1^3 a_3^1 \end{bmatrix}, \tag{76}$$

where the coefficients are defined as $a_i^j = \boldsymbol{G}_i \cdot \boldsymbol{G}_0^j$. Note that $\boldsymbol{G}_0^j$ denotes the covector $\boldsymbol{G}^j$ at the element center.

The selection of matrix $\tilde{\mathbf{M}}$ is not unique. Previous studies show that selection of different numbers and forms of EAS parameters have decisive effects on the computational cost and accuracy. Rah et al. [11] proposed a solid-shell element based on three EAS parameters and the ANS method, showing excellent convergence and performance, *i.e.*, passing both membrane and out-of-plane bending patch tests, free from thickness and transverse shear locking, and good precision under high element aspect ratios. Besides, investigations indicate that introducing additional four EAS parameters in the in-plane normal strain field can alleviate the volumetric locking. Here, we implement the EAS7ANS1 element, with the

corresponding seven EAS parameters expressed as [11]

$$\tilde{\mathbf{M}} = \begin{bmatrix} 0 & 0 & 0 & \xi^1 & \xi^1\xi^2 & 0 & 0 \\ 0 & 0 & 0 & 0 & 0 & \xi^2 & \xi^1\xi^2 \\ \xi^3 & \xi^1\xi^3 & \xi^2\xi^3 & 0 & 0 & 0 & 0 \\ 0 & 0 & 0 & 0 & 0 & 0 & 0 \\ 0 & 0 & 0 & 0 & 0 & 0 & 0 \\ 0 & 0 & 0 & 0 & 0 & 0 & 0 \end{bmatrix}. \tag{77}$$

To overcome shear locking effects induced by the compatible low-order interpolations, an assumed natural strain (ANS) interpolation on the compatible transverse shear strains is applied. For initially curved structures with geometric nonlinearity, curvature-thickness locking, also known as trapezoidal locking, would appear. This kind of locking effects can be eliminated by an ANS interpolation on the compatible transverse normal strain [5]. As shown in Fig. 4, a linear interpolation of the compatible transverse shear strains E_{13} and E_{23}, evaluated at the four sampling points (A, B, C and D) located at the four mid-points of the element edges, is applied,

$$\begin{bmatrix} \mathrm{E}_{13} \\ \mathrm{E}_{23} \end{bmatrix} = \frac{1}{2} \begin{bmatrix} (1-\xi^2)\,\mathrm{E}_{13}\,(\boldsymbol{\xi}_A) + (1+\xi^2)\,\mathrm{E}_{13}\,(\boldsymbol{\xi}_C) \\ (1-\xi^1)\,\mathrm{E}_{23}\,(\boldsymbol{\xi}_D) + (1+\xi^1)\,\mathrm{E}_{23}\,(\boldsymbol{\xi}_B) \end{bmatrix}, \tag{78}$$

where the coordinates of sampling points are $\boldsymbol{\xi}_A = (0,-1,0)$, $\boldsymbol{\xi}_B = (1,0,0)$, $\boldsymbol{\xi}_C = (0,1,0)$, $\boldsymbol{\xi}_D = (-1,0,0)$, and $\mathrm{E}_{13}\,(\boldsymbol{\xi}_{\mathrm{A}})$, $\mathrm{E}_{13}\,(\boldsymbol{\xi}_{\mathrm{C}})$, $\mathrm{E}_{23}\,(\boldsymbol{\xi}_{\mathrm{D}})$ and $\mathrm{E}_{23}\,(\boldsymbol{\xi}_{\mathrm{B}})$ denote transverse shear strains, respectively. The above interpolation on the transverse shear strains eliminates the shear locking problem, and allows for pure bending deformation without parasitic transverse shear strains.

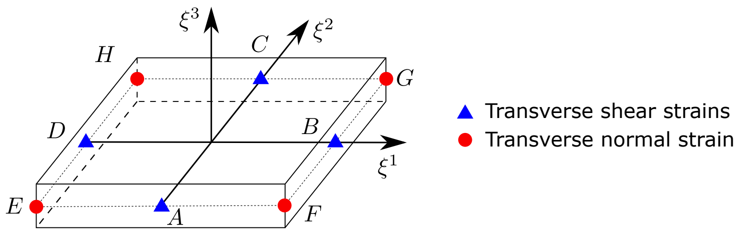

Supplementary Figure 4. Sampling points of ANS interpolation of an eight-node hexahedral element in isoparametric space. The blue triangles denote sampling points for transverse shear strains, while the red dots indicate sampling points for transverse normal strain.

For the transverse normal strains, a bilinear interpolation sampled at the four corners (E, F, G and H) of the element mid-surface is imposed,

$$\mathrm{E}_{33} = \sum_{i=1}^{4} N_i \left(\xi^1,\ \xi^2\right) \mathrm{E}_{33}\left(\boldsymbol{\xi}_i\right), \tag{79}$$

where $N_i = \left(1 + \xi_i^1 \xi^1\right)\left(1 + \xi_i^2 \xi^2\right)/4$, ξ_i^1 and ξ_i^2 are the coordinates of sampling points with $\boldsymbol{\xi}_1 = \boldsymbol{\xi}_E = (-1, -1, 0)$, $\boldsymbol{\xi}_2 = \boldsymbol{\xi}_F = (1, -1, 0)$, $\boldsymbol{\xi}_3 = \boldsymbol{\xi}_G = (1, 1, 0)$ and $\boldsymbol{\xi}_4 = \boldsymbol{\xi}_H = (-1, 1, 0)$. The above interpolation on transverse normal strain remedies the thickness locking effects.

The combination of EAS and ANS method can alleviate several locking effects mentioned above, improve the element convergence, enhance the mesh distortion insensitivity, and optimize the performance of solid-shell elements especially for the analyses of thin-walled and multilayered composite structures.

III. EXPERIMENTS

A. Characterization

Table 1 illustrates the changes in length L_t and width W_t of the LCE monolayer square sheets (50 mm × 50 mm × 1.2 mm) as temperature varies.

Supplementary Table 1. The deformed dimensions of square samples at different temperatures.

Temperature	65°C	70°C	75°C	80°C	85°C	90°C
L_t (mm)	51.17	51.58	51.82	52.36	52.75	53.08
W_t (mm)	49.22	48.91	48.27	47.64	46.78	45.96

B. Shape morphing of LCE bilayers

We provide in Figs. 5-8 the morphology evolutions of LCE strips corresponding to all the experimental data and simulations in Fig. 2 in the main text. In Fig. 5, the bending evolutions in experiments and simulations are given, where $\theta_u = 0°,\ \theta_l = 90°$. The bending angle of the strip grows with the increase of thermal load ΔT, and the deformed shape eventually approaches circular.

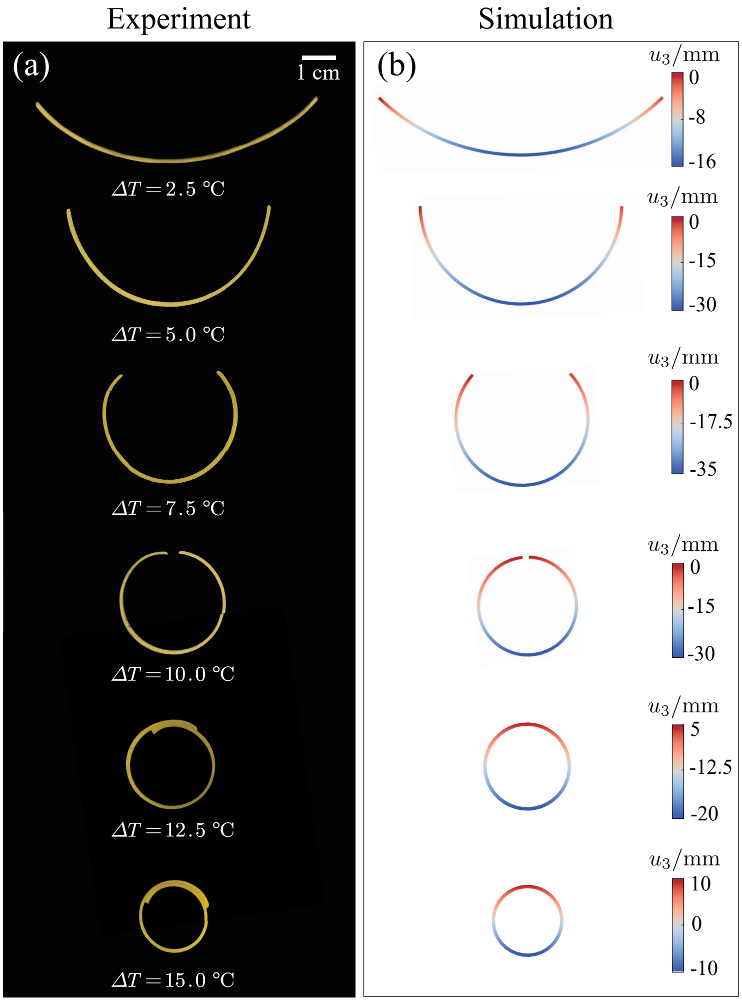

Supplementary Figure 5. Bending deformations of LCE bilayers under thermal loading: (a) Experimental results. (b) Simulations. The director orientations of bilayers take $\theta_u = 0°$ and $\theta_l = 90°$, respectively. The thermal load ΔT ranges from 2.5 °C to 15 °C.

Morphology selection of twisting deformation are listed in Figs. 6 and 7, which respectively correspond to Figs. 2(b) and 2(c) in the main text. In Fig. 6, the temperature difference in (b) remains $\Delta T = 15$ °C, while the director angle difference $\Delta\theta$ increases from 10° to 170°. When $\Delta\theta = 90°$, the twisting angle reaches its maximum value. Figure 7 suggests, both experimentally and numerically, that the twisting angle increases with the rise of thermal load ΔT.

Morphology evolution of spiral mode is given in Fig. 8, where $\theta_u = 120°$ and $\theta_l = 30°$. With the increase of thermal load, the spiral radius of the bilayer decreases, while the spiral number increases instead.

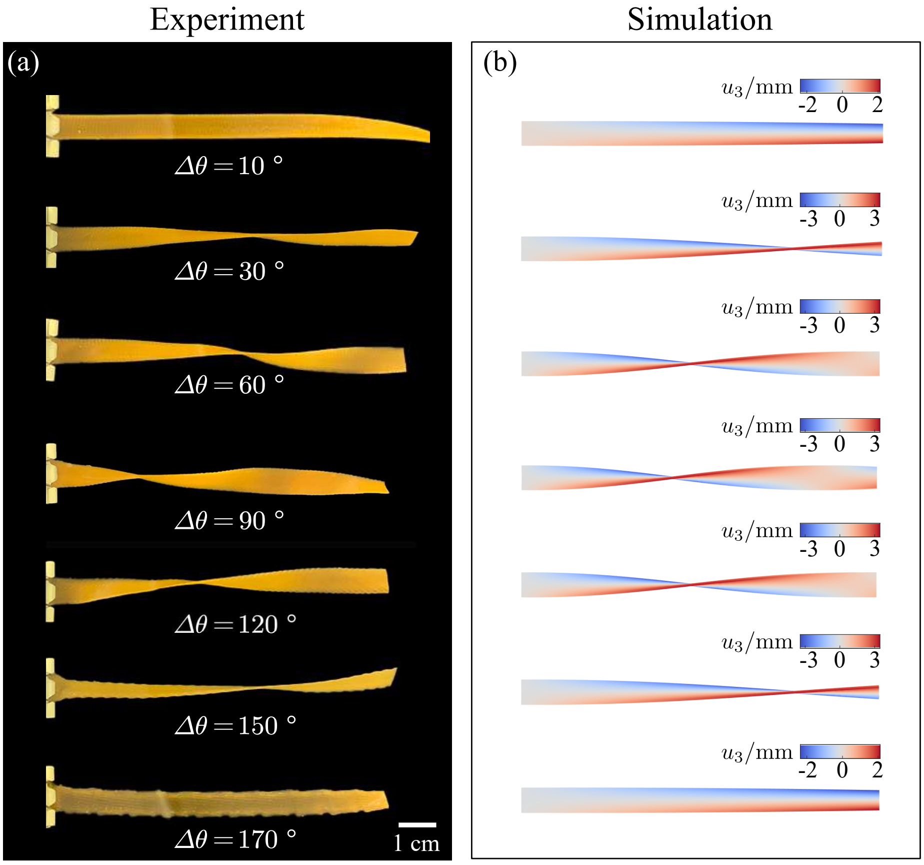

Supplementary Figure 6. Twisting modes of LCE bilayers with different director orientation angles under $\Delta T = 15$ °C: (a) Experimental results. (b) Simulations. The sum of director angles in bilayers satisfies $\theta_u + \theta_l = 180°$, with the angle difference $\Delta\theta = \theta_u - \theta_l$ ranging from 10° to 170°. When $\Delta\theta = 90°$, the twisting angle reaches its maximum value.

C. Fabrication of active "leaves"

Figure 9 illustrates the fabrication process of LCE "leaves" that can spontaneously deform adapted to environmental cues.

D. Experimental setup of droplet collection

As illustrated in Fig. 10, the droplet collection system comprises a frame, a rotating exhibition platform, and an irrigation water gun. The rotating platform was employed to simulate omnidirectional rainfall, rotating at a constant speed of 7 r/min.

In the examination of wind resistance performance of single strips (twisted or flat), the

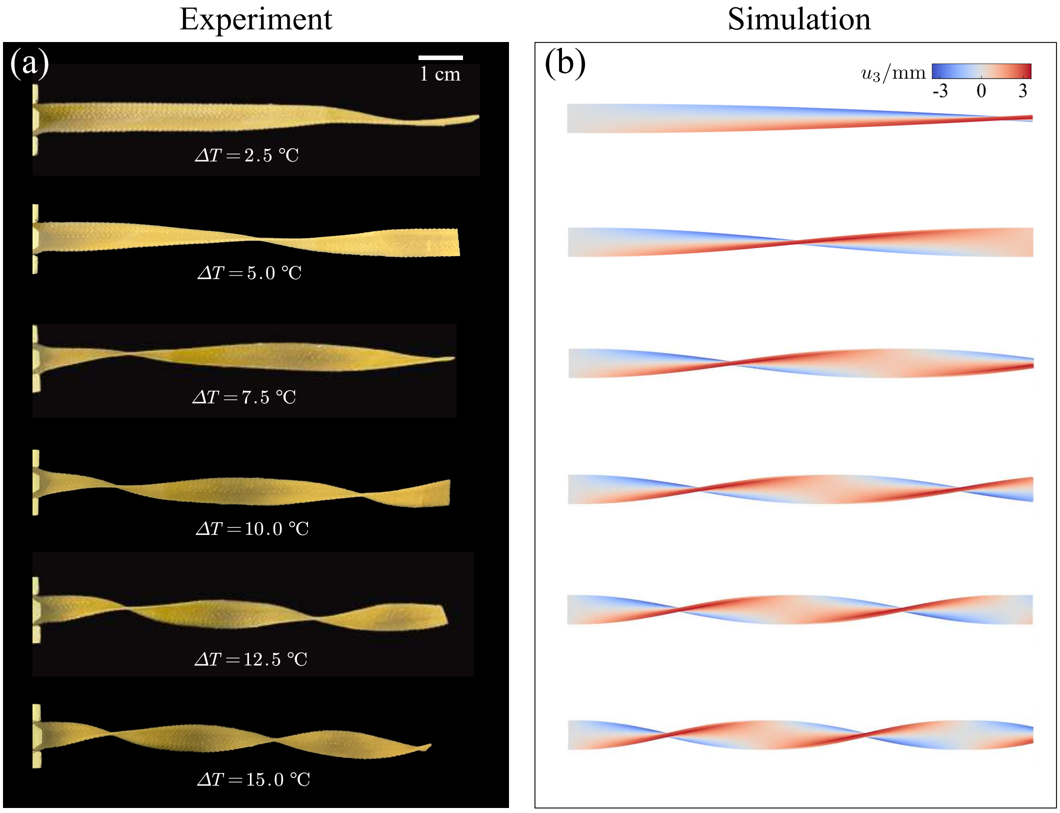

Supplementary Figure 7. Twisting morphology evolution of a LCE bilayer under thermal loading: (a) Experimental results. (b) Simulations. The director orientations of bilayers take $\theta_u = 135°$ and $\theta_l = 45°$, respectively. The thermal load ΔT ranges from 2.5 °C to 15 °C. The twisting deformation becomes increasingly significant with the rise of thermal load ΔT.

Supplementary Table 2. Data of droplet collection experiments.

Group	Configuration	Exp. 1	Exp. 2	Exp. 3	Exp. 4	Exp. 5	Average
Single "leaf"	Twisting	6.3 ml	6.0 ml	6.2 ml	6.3 ml	6.1 ml	6.2 ml
	Bending	3.0 ml	2.8 ml	3.2 ml	3.0 ml	2.8 ml	3.0 ml
Single-level clusters	Twisting	16.0 ml	15.5 ml	15.5 ml	14.0 ml	16.0 ml	15.4 ml
	Bending	8.5 ml	9.0 ml	8.5 ml	8.5 ml	6.5 ml	8.2 ml
Multi-level clusters	Twisting	31.2 ml	31.4 ml	31.2 ml	30.5 ml	32.0 ml	31.3 ml
	Bending	16.5 ml	16.5 ml	15.5 ml	16.0 ml	17.0 ml	16.3 ml
Windy environment	Twisting	10.0 ml	10.0 ml	10.5 ml	9.5 ml	9.8 ml	10.0 ml
	Flat	6.5 ml	6.5 ml	6.0 ml	6.5 ml	6.0 ml	6.3 ml

wind speed was set 5 m/s, measured by an anemometer. In the droplet collection experiments in windy environment, the wind speed was set 7 m/s. All droplet collection experiments were repeated for five times. The rainfall for the single "leaf" and single-level clusters lasted for 5 mins, while for multi-level clusters for 4 mins. Experimental data is listed in

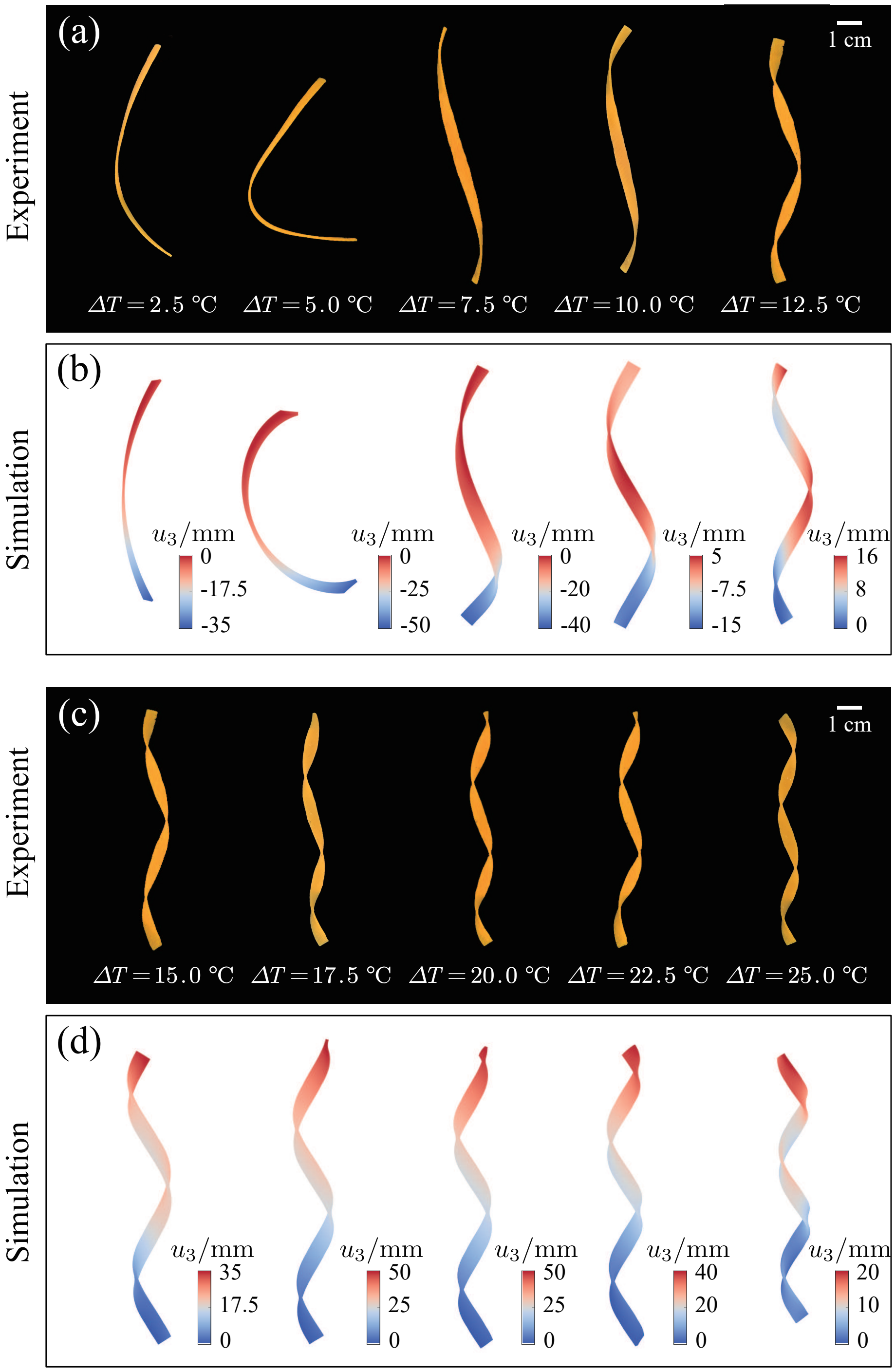

Supplementary Figure 8. Bending-twisting coupling spiral morphology evolution of LCE bilayers under thermal loading: (a) and (c) Experimental results. (b) and (d) Simulations. The director orientations of bilayers take $\theta_u = 120°$ and $\theta_l = 30°$, respectively. The thermal load ΔT ranges from 2.5 °C to 25 °C. With the rise of thermal load, the spiral radius reduces, while the spiral number dramatically increases.

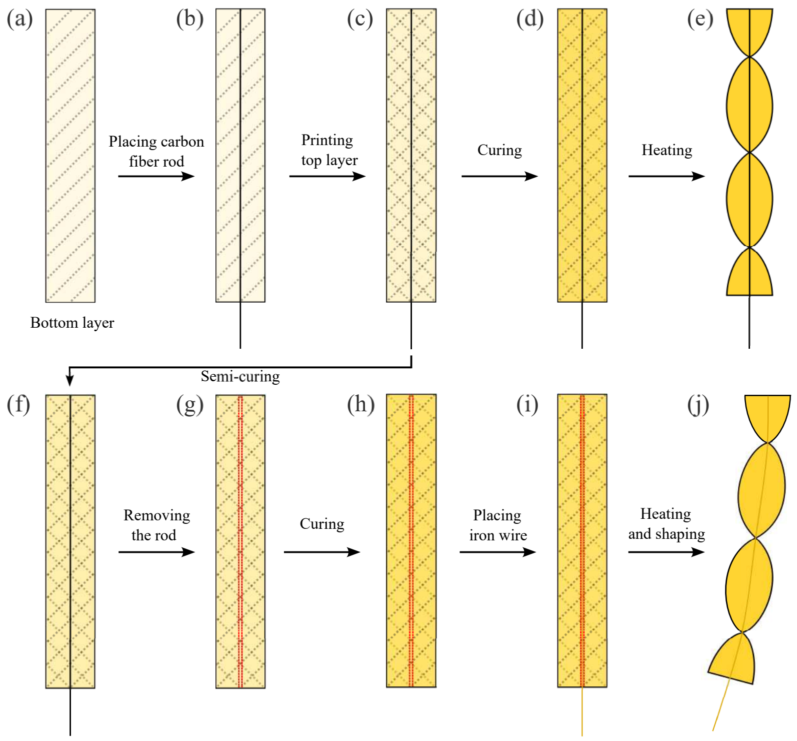

Supplementary Figure 9. Fabrication process of LCE "leaves": (a) Printing the bottom layer. (b) Placing a carbon fiber rod at the center. (c) Printing the upper layer to cover the rod. (d) Curing. (e) Heating the "leaf" to morph into a twisting shape. (f) Semi-curing. (g) Removing the carbon fiber from the "leaf". (h) Complete curing. (i) Insertion of an iron wire into the channel. (j) Twisting deformation of the "leaf" upon heating, which also allows for a global bending deformation.

Supplementary Table 2, which suggests that the twisting morphology, as opposed to the pure bending configuration, is significantly more effective in droplet collection (directing rainfall towards the root). Moreover, even in windy environments, the twisting shape dramatically enhances leaf stiffness which significantly contributes to droplet collection.

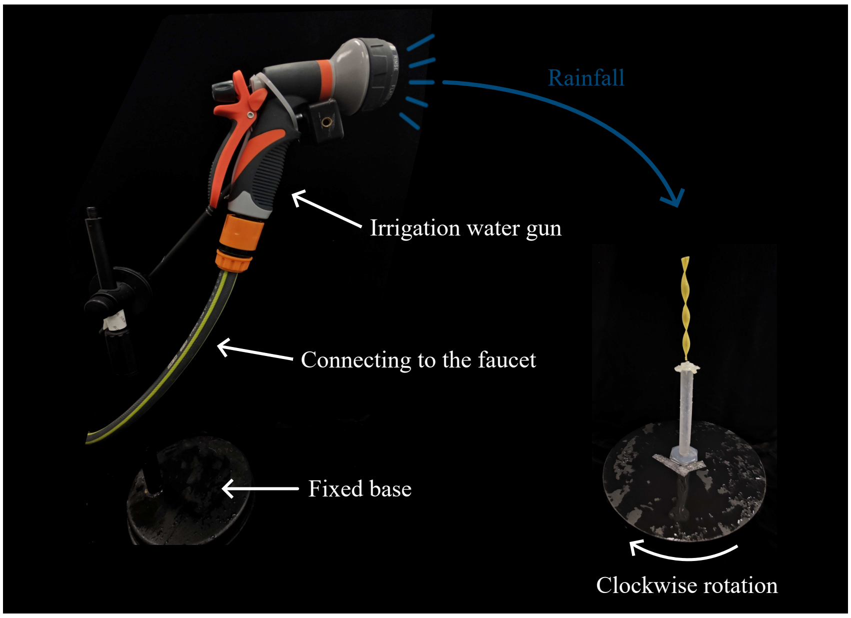

Supplementary Figure 10. Experimental setup of droplet collection.

[1] ABesheva, 2016. Pancratium maritimum. Image with CC BY-SA 4.0 license. Available at: `https://commons.wikimedia.org/w/index.php?curid=49434833` [Accessed 2 March 2025].

[2] Aharoni, H., Sharon, E., Kupferman, R., 2014. Geometry of thin nematic elastomer sheets. Phys. Rev. Lett. 113, 257801.

[3] Ambulo, C.P., Burroughs, J.J., Boothby, J.M., Kim, H., Shankar, M.R., Ware, T.H., 2017. Four-dimensional printing of liquid crystal elastomers. ACS Appl. Mater. Interfaces 9, 37332–37339.

[4] Armon, S., Efrati, E., Kupferman, R., Sharon, E., 2011. Geometry and mechanics in the opening of chiral seed pods. Science 333, 1726–1730.

[5] Betsch, P., Stein, E, 1995. An assumed strain approach avoiding artificial thickness straining for a non-linear 4-node shell element. Commun. Numer. Methods Eng. 11, 899–909.

[6] Chen, Z., Majidi, C., Srolovitz, D.J., Haataja, M., 2011. Tunable helical ribbons. Appl. Phys. Lett. 98, 011906.

[7] Efrati, E., Sharon, E., Kupferman, R., 2009. Elastic theory of unconstrained non-Euclidean plates. J. Mech. Phys. Solids 57, 762–775.

[8] McMaster, I., 2016. Persoonia helix. Image with CC BY 4.0 license. Available at: `https://`

images.ala.org.au/image/details?imageId=ffbac481-ed51-47f5-91f1-3638c867b009 [Accessed 2 March 2025].

[9] PlantNet, 2022. Ornithogalum concordianum, observation by sevilla ruby. Image with CC BY-SA 4.0 license. Available at: https://identify.plantnet.org/k-world-flora/observations/1014313931 [Accessed 2 March 2025].

[10] PlantNet, 2025. Albuca namaquensis, observation by bery pannkuk. Image with C-C BY-SA 4.0 license. Available at: https://identify.plantnet.org/k-world-flora/observations/1025574819 [Accessed 2 March 2025].

[11] Rah, K., Van Paepegem, W., Habraken, A.M., Degrieck, J., Alves de Sousa, R.J., Valente, R.A.F., 2013. Optimal low-order fully integrated solid-shell elements. Comput. Mech. 51, 309–326.

[12] Simo, J. C., Rifai, M. S., 1990. A class of mixed assumed strain methods and the method of incompatible modes. Int. J. Numer. Methods Eng. 29, 1595–1638.

[13] Struik, D.J., 1988. Lectures on Classical Differential Geometry. Dover Publications, 2nd edition.

[14] Timoshenko, S., 1925. Analysis of bi-metal thermostats. J. Opt. Soc. Am. 11, 233–255.

[15] van Rees, W.M., Vouga, E., Mahadevan, L., 2017. Growth patterns for shape-shifting elastic bilayers. Proc. Natl. Acad. Sci. USA 11, 11597–11602.

[16] Vu-Quoc, L., Tan, X.G., 2003. Optimal solid shells for non-linear analyses of multilayer composites. I. Statics. Comput. Methods Appl. Mech. Eng. 192, 975–1016.

[17] Zhao, Z.L., Zhao, H.P., Chang, Z., Feng, X.Q., 2014. Analysis of bending and buckling of pre-twisted beams: A bioinspired study. Acta Mech. Sin. 30, 507–515.
