# Supplementary material for: Active twisting for adaptive droplet collection
Source: Nat Comput Sci. 2025 Apr 21;5(4):313–21. doi: 10.1038/s43588-025-00786-w (PMC12021652; doi:10.1038/s43588-025-00786-w)
Supplement: Supplementary file 6 — MATLAB code. [file 43588_2025_786_MOESM6_ESM.zip › Code/README.pdf]

1. System requirements

The required software is Matlab R2018b or later version.

For Matlab system requirements and minimum configuration of computer hardware, please refer to:

https://ww2.mathworks.cn/support/requirements/matlab-system-requirements.html

2. Installation guide

For the installation of Matlab R2018b, please refer to the official website:

https://ww2.mathworks.cn/

3. Demo

For running Matlab code, please refer to 'video_Fig_2a.mp4' and 'video_Fig_3mode.mp4'. To reproduce the figures in the main text, the Matlab code runs in only 2-5 seconds. For calculating the deformation of the LCE bilayer under thermal loads, the runtime may vary from several minutes to half a day, depending on the number of elements and load magnitude.

4. Instruction for use

For the Matlab codes 'Fig_2a.m', 'Fig_2b.m', 'Fig_2c.m', and 'Fig_2d.m', open each file in Matlab R2018b. Simply click 'Run' to execute the code, and four figures corresponding to Fig. 2(a)-(d) in the main text will be generated. These figures incorporate theoretical, experimental and simulation data.

For the MATLAB code 'Fig_3phase.m', click 'Run' to generate the phase diagram corresponding to Fig. 3 in the main text. For code 'Fig_3mode.m', click 'Run Section' for the sections corresponding to bending, twisting and spiral modes to read simulation data and generate deformation contour for these three modes as shown in Fig. 3.

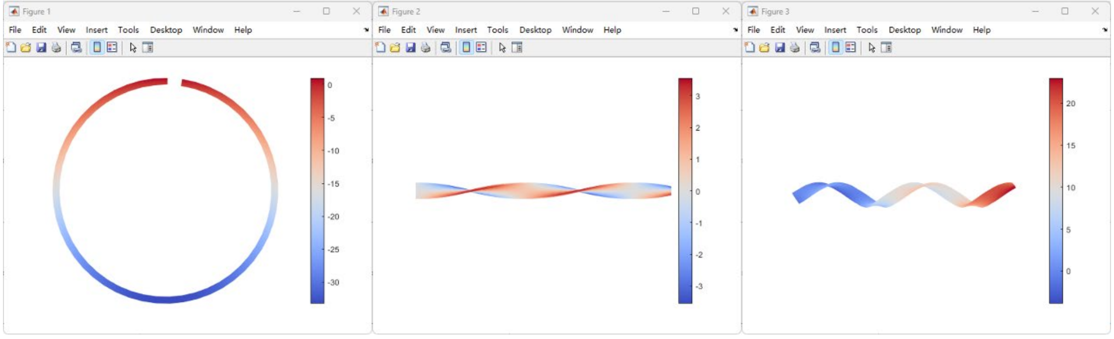

For the Matlab code 'Fig3.m', click 'Run' to simulate the deformation of an LCE bilayer using the solid-shell model, which corresponds to the morphologies shown in Fig. 3 of the main text and in Figs. S7-S10 in the supplementary. Before running the program, you may adjust the following parameters:

Geometry and mesh parameters

- Dimensions: Length (L), Width (W), and Thickness (H).
- Number of elements in each direction: Length (Le), Width (We), and Height (He).

Material and load parameters

- dT: The final temperature change applied in the simulation.
- alpha_per: Expansion factor in the perpendicular direction.
- alpha_par: Expansion factor in the parallel direction.
- Director angle of lower layer: theta1 (in-plane angle) and phi1 (out-of-plane angle).
- Director angle of upper layer: theta2 (in-plane angle) and phi2 (out-of-plane angle).
- IncFac: The initial load increment factor.
- TOL: The convergence tolerance for each step.

Results and post-processing

Upon completion, the program will automatically display a deformation contour for the selected calculation step, visualizing the deformation based on your defined parameters.

- istp: Indicates the selected step at which results are displayed.
- UU(1, istp): Gives the load factor at step 'istp', with temperature change at this step as dTi = dT*UU(1, istp).
- UU(2:end, istp): Represents the solution at step 'istp'.
